# Hysterectomy Rate Following Endometrial Ablation in Ontario: A Cohort Analysis of 76,446 Patients

**DOI:** 10.52054/FVVO.16.3.028

**Published:** 2024-09-30

**Authors:** J McGee, A McClure, S Ilnitsky, A Vilos, B Abu-Rafea, G Vilos

**Affiliations:** Western University, London, Ontario, Canada, N6A5W9; London Health Sciences Centre, London, Ontario, Canada, N6A5W9

**Keywords:** Endometrial ablation, hysterectomy, repeat ablation, myomectomy

## Abstract

**Background:**

Endometrial Ablation (EA) is an alternative to hysterectomy for the management of abnormal uterine bleeding (AUB); however, it does not eliminate the need for future surgical re-intervention.

**Objectives:**

The primary objective of this study was to establish long-term clinical outcomes including the risk of hysterectomy in women who had undergone a primary EA.

**Materials and Methods:**

This is a retrospective population-based cohort study utilising administrative data from the Canadian province of Ontario. This study assesses patients undergoing surgery in a publicly funded health care system.

**Main outcome measures:**

We assessed women in Ontario undergoing a primary EA over a 15-year period. The primary outcome was hysterectomy within 5 years of primary EA. Secondary outcomes included myomectomy and repeat EA. All outcomes were also reported for 1, 3, 5, 10 and 15 years of follow-up. Logistic regression was used to establish predictors of hysterectomy within 5 years of primary EA.

**Results:**

A total of 76,446 primary EAs were evaluated from 2002-2017, with 16,480 (21.56%) undergoing a subsequent surgical intervention. The average age of primary EA was 43.8 (+/- 6.3) years. Within 5 years, the evaluable cohort was 52,464, with 8,635 (16.46%) of women having proceeded to hysterectomy, 664 (1.27%) to myomectomy, and 2,468 (2.8%) to repeat ablation. By 15-years follow-up, the evaluable cohort was 1,788, with 28.75% had undergone a hysterectomy, 2.01% a myomectomy, and 5.20% a repeat EA. On logistic regression analysis, advancing age at time of EA was associated with significantly decreased odds of hysterectomy (OR=0.94, 95% CI 0.935-0.944, p<.0001) as was increasing surgical experience (OR=0.997, 95% CI 0.994-1.000, p=.022). Conversely, complex diagnosis (OR=1.102, 95% CI 1.042-1.164, p<.0001) and previous abdominal surgery (OR=1.288, 95% CI 1.222-1.357, p<0.0001) were associated with increased risk of subsequent hysterectomy.

**Conclusion:**

Primary EA is associated with a high risk of progression to subsequent hysterectomy or other surgical intervention, without evidence of plateau of risk with long term follow-up.

**What is new?:**

This study has the longest follow-up assessing hysterectomy outcomes in women undergoing a primary EA, with 28.75% of women having undergone a hysterectomy within 15 years of their EA.

## Introduction

Endometrial ablation (EA) is a minimally invasive procedure used to treat abnormal uterine bleeding (AUB), offered as a surgical alternative to hysterectomy. We previously reported on the safety of the procedure, reporting a low overall complication rate of 4.8% (Ilnitsky et al., 2021). Less firmly established however is the risk of repeat EA or subsequent hysterectomy in women undergoing the procedure.

While several studies have shown an increased risk of progression to subsequent surgical intervention including hysterectomy, these studies suffer from short term follow-up.

### Objective

The primary outcome was hysterectomy within 5 years of primary EA. Secondary outcomes included myomectomy and repeat EA. All outcomes were also reported for 1, 3, 5, 10 and 15 years of follow-up. Logistic regression was used to establish predictors of hysterectomy within 5-years of primary EA.

## Materials and methods

The methodology has been described elsewhere, (Ilnitsky et al., 2021) however we will briefly review here.

### Study design and data sources

This retrospective population-based cohort study included all women who underwent EA in the province of Ontario, Canada (population approximately 14 million), from October 1, 2002 to September 30, 2017. Table I presents the study exclusion criteria and number of patients excluded at each step of the cohort build.

**Table I:** Cohort exclusions.

Exclusion Criteria	Number Excluded	Number Included
Data Cleaning	40	95,714
Non-Ontario Resident	35	95,679
Age <18 or >105	36	95,643
No matching Ontario Health Insurance Plan record	12,733	82,910
History of endometrial/ovarian cancer	226	82,684
Ineligible concurrent procedure	5,030	77,654
Previous endometrial ablation	1,208	76,446

Note: Data cleaning includes: male sex and invalid age or date of death. Ineligible concurrent procedures include procedures related to the destruction/excision of the ovaries, uterus, or fallopian tubes, occlusion or dilation of the fallopian tubes, and implantation of an IUD or brachytherapy applicator.

In the current study, we used the Canadian Institute for Health Information’s (CIHI) Discharge Abstract Database, Same Day Surgery, and National Ambulatory Care Reporting System databases to obtain data related to hospitalisation, same day surgery services, and emergency department visits. We also used the Ontario Health Insurance Plan (OHIP) database to identify physician services and the Ontario Cancer Registry to determine cancer history. Additional patient and physician characteristics were obtained from the Registered Persons Database and the Institute for Clinical Evaluative Sciences (ICES) Physician Database, respectively. Within ICES, there is no ability to discern the type of EA a patient has undergone (including type of EA (resection vs thermal ablation) and which generation of technology was employed. In addition, the administrative data does not allow for a direct correlation as to why a patient underwent a given procedure. These datasets were linked using unique encoded identifiers and analysed at ICES Western. Reporting of this study follows the RECORD statement (see Table SI) (Benchimol et al., 2015).

### Baseline variables

Patient baseline characteristics are provided, including patient age, Charlson Comorbidity Index score, morbid obesity, history of diabetes or hypertension, and previous abdominal surgery or tubal ligation. We also captured the number of hospital admissions, gynaecology visits, and general practitioner visits during the previous 1-year period as indicators of healthcare utilisation. The following variables related to the procedure, institution, and surgeon are also reported: diagnosis/ indication for endometrial ablation (where more than one preoperative diagnosis was found, the indication for surgery was considered complex), anaesthetic classification (healthy/mild disease [ASA 1-2] vs severe systemic disease [ASA 3-5]), admission to hospital, institution teaching status (academic vs. community hospital), fiscal year, and surgeon sex and experience (years since medical school graduation). Annual EA volume (based on OHIP billings) for both the surgeon and the hospital are also reported.

### Statistical analysis

Descriptive statistics are provided for each baseline variable. The number and percentage of patients experiencing each outcome at each follow-up point (1, 3, 5, 10, and 15 years) is reported for patients who were followed for that entire outcome window. For example, 15-year outcomes are only reported for patients whose primary EA occurred at least 15-years before the study end date and who were not lost to follow-up (death or emigration) during the 15-year period. Patients undergoing an EA or myomectomy were not removed from the cohort and were evaluated for hysterectomy as long as they remained in the cohort.

Logistic regression was used to evaluate potential predictors of hysterectomy within 5 years of primary EA. The following covariates were included in the regression model: patient age, indication for surgery (complex [defined as an indication of bleeding plus another diagnosis: fibroids, pain, hyperplasia] vs bleeding only), previous abdominal surgery (including tubal ligation, 481 abdominal/ pelvic procedures were evaluated), anaesthetic classification (ASA <3 vs ASA 3+), and surgeon years of experience. Patients were excluded listwise from this analysis if they were missing data for any of the included covariates. All analyses were performed using SAS EG version 7.15 (SAS Institute, Cary, NC, USA).

### Main Outcome Measures

The primary outcome was hysterectomy within 5 years of primary EA. Secondary outcomes included repeat EA and myomectomy. All outcomes were reported for 1, 3, 5, 10, and 15 years of follow-up. Interventions were classified as either a repeat EA, myomectomy, or hysterectomy. All of the codes used to define these outcomes, as well as all other codes used in this study, are presented in Table SII. Of note, subsequent procedures may have been performed by gynaecologists not involved in the original surgery.

## Results

The cohort initially included 95,754 patients, but following 19,308 exclusions, the final cohort comprised 76,446 patients (Table I). The baseline characteristics of women undergoing a primary EA are presented in Table II. The average age of women was 43.8+/-6.3, with comorbidities of diabetes (6.6%), hypertension (17.6%) and morbid obesity (7.1%), and an ASA of 3+ (17%) seen throughout the cohort. The majority (94%) of women had AUB as their indication for surgery, while 28.5% had a complex preoperative diagnosis (signifying more than one preoperative diagnosis).

**Table II:** Baseline characteristics.

Variable		Total (n = 76,446)
Age (years)		43.8 ± 6.3
Rural		12,837 (16.8%)
Neighbourhood income		
	Quintile 1	11,832 (15.5%)
	Quintile 2	14,246 (18.6%)
	Quintile 3	15,971 (20.9%)
	Quintile 4	17,396 (22.8%)
	Quintile 5	16,824 (22.0%)
	Missing	177 (0.2%)
Comorbidities		
	Diabetes	5,092 (6.7%)
	Hypertension	13,426 (17.6%)
	Obesity	5,404 (7.1%)
ASA 3+		12,981 (17%)
Previous abdominal-pelvic surgery		28,015 (36.6%)

Note: Diagnoses are not mutually exclusive. Rural refers to people living in an area with less than 50,000 people. Quintile 1 refers to the lowest neighbourhood quintile range, Quintile 5 is the highest. ASA is the American Society of Anesthesiologists physical classification system, where ASA 3+ anaesthesia association refers to a patient with at least severe systemic disease limiting activity, or worse.

**Figure 1:**
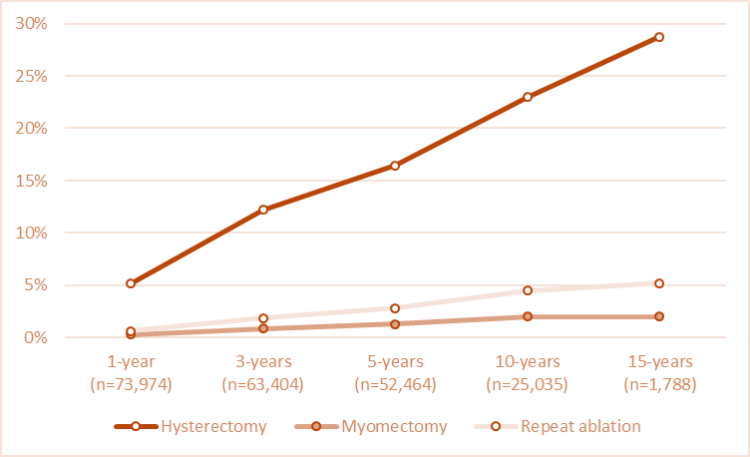
Surgical intervention over time.

We present surgical outcomes following primary EA in Table III. Of the 76,446 women evaluable, 16,480 (21.56%) went on to have a subsequent surgical intervention. Within one year of their EA, 73,974 patients remained in the cohort, with 3,818 (5.16%) having undergone hysterectomy, 981(1.28%) myomectomy, and 470 (0.64%) repeat ablation. At 5 years, the evaluable cohort was 52,464, with 8,635 (16.46%) having had a hysterectomy, 664 (1.27%) a myomectomy, and 2,468 (2.8%) a repeat ablation. With 10 years follow-up, the evaluable cohort was 25,035, with 5767 (22.99%) of women undergoing a hysterectomy, 504 (2.01%) a myomectomy, and 1,120 (4.47%) a repeat EA. By 15 years follow-up, the cohort had decreased to 1,788, with 514 (28.75%) of women undergoing a hysterectomy, 36 (2.01%) a myomectomy, and 93 (5.20%) a repeat EA. While the percentage of patients with hysterectomy increases with follow-up duration, the majority of patients undergo hysterectomy within the first few years after EA. For those women with 15-years of follow-up, 50% of those who have a hysterectomy do so within 2.5 years of having their EA, while 75% do so in just over 5 years. Surgical intervention over time is presented in Figure 1.

**Table III:** Surgical outcomes following primary endometrial ablation.

Follow-up time period (n= size of evaluable cohort)	Hysterectomy	Myomectomy	Repeat Ablation	Death or emigration
Within 1-year (n=73,974)	3,818 (5.16%)	228 (0.31%)	470 (0.64)	84 (0.11%)
Within 3-years (n=63,404)	7,736 (12.2%)	540 (0.85%)	1,195 (1.88%)	731 (1.15%)
Within 5-years (n=52,464)	8,635 (16.46%)	664 (1.27%)	1,468 (2.80%)	1,069 (2.04%)
Within 10-years (n=25,035)	5,756 (22.99%)	504 (2.01%)	1,120 (4.47%)	1,106 (4.42%)
Within 15-years (n=1,788)	514 (28.75%)	36 (2.01%)	93 (5.20%)	144 (8.05%)

We conducted a logistic regression (Table IV) including 49,693 women, looking at factors predictive of hysterectomy at 5 years. Advancing age at primary EA was associated with a 6% decrease in the odds of hysterectomy (OR=0.94, 95% CI 0.935-0.944, p<0.0001) per year of life. As compared to patients without a complex diagnosis, women with a surgical diagnosis beyond simply bleeding had a 10.2% increase in the odds of progressing to hysterectomy (OR = 1.102, 95% CI 1.042 - 1.164, p<0.0001). Previous abdominal surgery carried a 28.8% increase in odds of hysterectomy (OR=1.288, 95% CI 1.222 – 1.357, p<0.0001). Surgeon experience was a negative predictor of progression to hysterectomy, with 3% decreased odds with each additional year of age of the operator (OR=0.997, 95% CI 0.994-1.000, p<0.0001).

**Table IV:** Logistic regression predicting hysterectomy within 5 years of endometrial ablation. (n=49,653).

Variable	Odds Ratio	Lower 95%CI	Upper 95% CI
Age	0.940	0.935	0.944
Diagnosis (Complex vs Bleeding)	1.102	1.042	1.164
Previous abdominal surgery (yes vs no)	1.288	1.222	1.357
ASA 3+ (yes vs no)	1.022	0.954	1.095
Surgeon Experience	0.997	0.994	1.000

## Discussion

Women undergoing EA have a significant risk of progression to further surgical intervention, with hysterectomy the procedure of choice. Of the entire cohort, 21.56% ultimately received a hysterectomy as definitive treatment for their abnormal uterine bleeding (AUB). Within one-year post EA, 5.16% of women will have undergone hysterectomy, increasing to 16.46% at 5 years, 22.99% at 10 years, and 28.75% at 15 years. Furthermore, the slope of increase in hysterectomy rate shows no evidence of plateau at 15 years. Younger age was associated with a 6% increase in risk of hysterectomy per year of life. The average age was 43.8 for primary EA in this study, so that each year earlier for primary EA was associated with a 6% increase in risk of hysterectomy later in life, with each year later associated with a 6% decrease in risk of hysterectomy. A complex diagnosis as reason for an EA conferred a 10.2% increase in risk of hysterectomy. Previous abdominal surgery (including laparoscopic tubal ligation) was associated with a 28.8% risk of hysterectomy. With increasing surgeon experience (using age as a surrogate), the likelihood of requiring a subsequent hysterectomy decreased by 3% for each increase in year of experience.

In a longitudinal study looking at surgical outcomes following EA in women enrolled with Kaiser Permanente with up to 8 years of follow-up, 21% underwent a subsequent hysterectomy, while 3.9% were treated with a uterine conserving procedure (either myomectomy or repeat EA). Regression analysis showed age to be the most significant predictor of hysterectomy, with women < 45 years of age at the time of EA having 2.1 times the risk of progressing to hysterectomy, with >40% of women <40 years of age at time of EA subsequently undergoing hysterectomy. Furthermore, there was no evidence of plateau of risk even at 8 years of follow-up, where the hysterectomy risk was 26% (Longinotti et al., 2008). These findings were echoed in a systematic review (Beelen et al., 2019). A cohort study from the UK of 114,910 women who had undergone EA (by all methods) for AUB between January 2000 through December 2011 showed an associated risk of hysterectomy after the initial ablation at 1, 2 and 5 years were 5.6%, 9.6%, and 13.5%, respectively (Bansi-Matharu et al., 2013). The authors also reported higher rates of subsequent surgery associated with younger age at initial EA, with women aged under 35 years having an adjusted hazard ration of 2.83 (95% CI: 2.67- 2.99) (Bansi-Matharu et al., 2013). The Mistletoe study evaluated hysterectomy outcomes at 4 to 5 years post EA, with 16% of the cohort having progressed to hysterectomy (Overton et al., 1997). Oderkerk et al. (2023) published a meta-analysis and systematic review showing an increase in risk of hysterectomy following EA from 4.3% at 1 year to 12.4% at 5 years.

Two cohort studies evaluated hysterectomy after EA utilising multivariable analysis, showing increased risk with younger age at EA, prior tubal ligation, and a diagnosis of preoperative dysmenorrhea (Longinotti et al., 2008; El-Nashar et al., 2009). Our results mirror the published literature, showing an increase in surgical intervention over time with no evidence of plateau or regression of risk.

The large number of women progressing to hysterectomy following a primary EA is sobering and suggests that EA for the broader patient population, rather than being a definitive management strategy, is best considered an intervention capable of temporising symptomatology until ultimate surgical treatment can be undertaken. Consequently, if an EA is to be performed, clinicians should rethink the long-term outcomes and cost-effectiveness of this procedure. Serious consideration and further exploration should be given towards combination treatments such as EA concomitantly with a levonorgestrel intrauterine system (LNG-IUS) or a single injection of 150 mg of medroxyprogesterone acetate (DMPA), as has been reported in some preliminary studies (Vaughan and Byrne, 2012; Vilos et al., 2013; Oderkerk et al., 2021). Such an approach has potential to extend the time period, perhaps into natural menopause, before further treatment is needed and possibly eliminate the need for additional therapy including hysterectomy.

The association between previous abdominal surgery and subsequent hysterectomy warrants further exploration. Our study showed that previous abdominal surgery had the largest association with progression to hysterectomy, however we were unable to determine the reason for hysterectomy at the individual level, and potentially severity of symptomatology is the explanation. Included in our ascertainment of previous abdominal surgery was tubal ligation, a procedure attributed to post-ablation syndrome with increased cyclic pain. Tubal ligation has a reported 4-5x increased risk of hysterectomy over vasectomy in a study comparing women adopting these forms of permanent contraception (Hillis et al., 1998). In addition to severity of symptomatology associated with the cyclic pain described in post-ablation syndrome, we propose that patients may become socialised to surgical intervention or in some way see themselves as appropriate candidates for hysterectomy following an EA. This has been argued in the setting of hysterectomy post tubal ligation, and a similar logic follows for post-EA patient, so that surgical intervention becomes an increasingly tenable option when there is a history of previous abdominal surgery (Pollack, 2003).

Our study also showed an association between surgeon experience and a decreased risk of subsequent hysterectomy. Surgeon experience has been found in other literature to be predictive of better outcomes. Increased surgeon experience may translate into better outcomes in two ways: firstly, it may relate to improve/refined surgical technique thereby minimising AUB post procedure; secondly, it may reflect better assessment and patient selection for an EA at the outset.

The strength of our study is the large cohort size and consistent methodology throughout the long- term follow-up. We report real world outcomes that align with outcomes previously reported in cohort and randomised control trials.

Our study has limitations. As mentioned, we were unable to determine cause of ‘failure’ or reason why patients proceeded to another surgical intervention. In addition, we could not differentiate between EA type or generation of EA device, however studies are conflicting in terms of whether this is predictive of progression to hysterectomy (Longinotti et al., 2008; Shavell et al., 2012).

## Conclusion

Endometrial ablation is a commonly performed gynaecologic procedure used to address AUB. Our analysis shows no evidence of plateau of risk of proceeding to hysterectomy, with 16.46% of women undergoing hysterectomy at 5 years, 22.99% of women undergoing hysterectomy at 10 years, and 28.75% of women undergoing hysterectomy by 15 years of follow-up. We identified important factors that reflect (surgeon experience and older patient age at time of EA) and increase (complex diagnosis and previous abdominal surgery) associated risk of hysterectomy at 5 years. Our study represents the largest cohort of patients with the longest follow-up published to date, addressing the question of surgical interventions post primary EA. The database sources are robust and reflect real world application of EA, making this analysis generalisable to other jurisdictions. Our analysis identifies unique covariates contributing to the success of EA. This study provides valuable information clinicians can utilise when counselling patients about EA versus another more definitive surgery.

## Supplementary material

Table SI RECORD statement checklist.

Table SII Intervention and diagnostic codes.
