# Supplementary material for: Hysterectomy Rate Following Endometrial Ablation in Ontario: A Cohort Analysis of 76,446 Patients
Source: Facts Views Vis Obgyn. 2024 Sep 30;16(3):311–6. doi: 10.52054/FVVO.16.3.028 (PMC11569427; doi:10.52054/FVVO.16.3.028)
Supplement: Table SI — RECORD statement checklist. [file FVVinObGyn-16-311-st001.pdf]

Table SI. - RECORD statement checklist.

Item	Description	Location reported (Line)
Title and abstract	a) Indicate the study's design with a commonly used term in the title or the abstract	2
	b) Provide in the abstract an informative and balanced summary of what was done and what was found	71-91
	c) The type of data used should be specified in the title or abstract. When possible, the name of the databases used should be included*	373-74
	d) If applicable, the geographic region and timeframe within which the study took place should be reported in the title or abstract* lines	74
	e) If linkage between databases was conducted for the study, this should be clearly stated in the title or abstract* not reported	Not reported (NR)
Background rationale	Explain the scientific background and rationale for the investigation being reported	102-114
Objectives	State specific objectives, including any prespecified hypotheses	123-126
Study Design	Present key elements of study design early in the paper	158-164
Setting	Describe the setting, locations, and relevant dates, including periods of recruitment, exposure, follow-up, and data collection	128-140
Participants	a) Give the eligibility criteria, and the sources and methods of selection of participants. Describe methods of follow-up	123-125
	b) The methods of study population selection (such as codes or algorithms used to identify subjects) should be listed in detail. If this is not possible, an explanation should be provided*	125-126, 383
	c) Any validation studies of the codes or algorithms used to select the population should be referenced. If validation was conducted for this study and not published elsewhere, detailed methods and results should be provided*	NR
	d) If the study involved linkage of databases, consider use of a flow diagram or other graphical display to demonstrate the data linkage process, including the number of individuals with linked data at each stage*	383
Variables	a) Clearly define all outcomes, exposures, predictors, potential confounders, and effect modifiers. Give diagnostic criteria, if applicable.	144-154

Item	Description	Location reported (Line)
	b) A complete list of codes and algorithms used to classify exposures, outcomes, confounders, and effect modifiers should be provided. If these cannot be reported, an explanation should be provided*	404
Data sources/ measurement	For each variable of interest, give sources of data and details of methods of assessment (measurement). Describe comparability of assessment methods if there is more than one group	144-154
Bias	Describe any efforts to address potential sources of bias	166-171
Study size	Explain how the study size was arrived at	383
Quantitative variables	Explain how quantitative variables were handled in the analyses. If applicable, describe which groupings were chosen, and why	158-173
Statistical methods	a) Describe all statistical methods, including those used to control for confounding	158-173
	b) Describe any methods used to examine subgroups and interactions	NR
	c) Explain how missing data were addressed	171-175
	d) If applicable, explain how loss to follow-up was addressed	NR
	e) Describe any sensitivity analyses	NR
Data access and cleaning methods	a) Authors should describe the extent to which the investigators had access to the database population used to create the study population*	185-186
	b) Authors should provide information on the data cleaning methods used in the study*	NR
Linkage	State whether the study included person-level, institutional-level, or other data linkage across two or more databases. The methods of linkage and methods of linkage quality evaluation should be provided*	144-154
Participants	a) Report the numbers of individuals at each stage of the study	383
	b) Give reasons for non-participation at each stage.	383
	c) Consider use of a flow diagram	383
	d) Describe in detail the selection of the persons included in the study (i.e., study population selection) including filtering based on data quality, data availability and linkage. The selection of included persons can be described in the text and/or by means of the study flow diagram*	383
Descriptive data	a) Give characteristics of study participants and information on exposures and potential confounders	388
	b) Indicate the number of participants with missing data for each variable of interest	383
	c) Summarise follow-up time	220-225

Item	Description	Location reported (Line)
Outcome data	Report numbers of outcome events or summary measures over time	222-225
Main results	a) Give unadjusted estimates and, if applicable, confounder-adjusted estimates and their precision (e.g., 95% confidence interval). Make clear which confounders were adjusted for and why they were included	208-216
	b) Report category boundaries when continuous variables were categorised	388
	c) If relevant, consider translating estimates of relative risk into absolute risk for a meaningful time period	NR
Other analyses	Report other analyses done—e.g., analyses of subgroups and interactions, and sensitivity analyses	NR
Key results	Summarise key results with reference to study objectives	
Limitations	a) Discuss limitations of the study, taking into account sources of potential bias or imprecision. Discuss both direction and magnitude of any potential bias	220-235
	b) Discuss the implications of using data that were not created or collected to answer the specific research question(s). Include discussion of misclassification bias, unmeasured confounding, missing data, and changing eligibility over time, as they pertain to the study being reported*	NR
Interpretation	Give a cautious overall interpretation of results considering objectives, limitations, multiplicity of analyses, results from similar studies, and other relevant evidence	307-318
Generalisability	Discuss the generalisability (external validity) of the study results	296-298
Funding	Give the source of funding and the role of the funders for the present study and, if applicable, for the original study on which the present article is based	14-16
Accessibility of protocol, raw data, and programming code	Authors should provide information on how to access any supplemental information such as the study protocol, raw data, or programming code*	NA

Note: n/a = not applicable; *RECORD statement addition to the STROBE statement.
