# Supplementary material for: Hysterectomy Rate Following Endometrial Ablation in Ontario: A Cohort Analysis of 76,446 Patients
Source: Facts Views Vis Obgyn. 2024 Sep 30;16(3):311–6. doi: 10.52054/FVVO.16.3.028 (PMC11569427; doi:10.52054/FVVO.16.3.028)
Supplement: Table SII — Intervention and diagnostic codes. [file FVVinObGyn-16-311-st002.pdf]

Table SII. - Intervention and diagnostic codes.

Variable	Code type	Codes
Endometrial ablation	Feecode	S772
	CCI	1RM59BAAG, 1RM59BAAK, 1RM59BABD, 1RM59BACG, 1RM59BAEA, 1RM59BAGX, 1RM59CAAD, 1RM59CAAG, 1RM59CAAK, 1RM59CAAW, 1RM59CABD, 1RM59CACG, 1RM59CAEA, 1RM59CAGX
	CCP	8019
Diagnosis: Bleeding	ICD-10	N920, N921, N924, N925, N926, N930, N938, N939, N948, N949, N950
Diagnosis: Fibroids	ICD-10	88900, D250, D251, D252, D259, D267, D269
Diagnosis: Hyperplasia	ICD-10	N850, N851, N852
Diagnosis: Pain	ICD-10	N800, N801, N802, N803, N804, N805, N808, N809, N941, N944, N945, N946
Diagnosis: Other	ICD-10	N840, N841, N848, N849, N856, N857, N858, N859, N882
Endometrial or Ovarian Cancer	ICD-0-3	C56, C541
Obesity	FEE	E010, E676
	ICD-10	E660, E661, E662, E668, E669
	ICD-9	2780, 2781
Gynaecology visit	Feecode	A203, A204, A205, A206, C203, C204, C205, C206, W305, W306
ASA 3+	Feecode	E016, E017, E022
Previous Abdominal surgery	Feecode	P018, P020, P027, P028, P036, P039, P041, P042, P045, P046, R708, R783, R784, R785, R786, R802, R805, R806, R811, R814, R817, R824, R828, R834, R839, R852, R858, R859, R860, R861, R877, R879, R885, R934, R937, S149, S150, S151, S154, S155, S156, S157, S158, S159, S160, S162, S164, S165, S166, S167, S168, S169, S170, S171, S172, S173, S174, S175, S176, S177, S180, S182, S183, S184, S185, S187, S188, S189, S191, S192, S193, S194, S195, S196, S197, S199, S201, S202, S204, S205, S206, S213, S214, S215, S216, S217, S218, S222, S223, S225, S226, S227, S228, S229, S231, S241, S242, S243, S246, S247, S248, S249, S251, S256, S257, S258, S259, S310, S312, S313, S314, S315, S316, S317, S318, S319, S321, S325, S329, S330, S332, S340, S343, S344, S434, S435, S437, S438, S440, S441, S442, S443, S444, S445, S446, S447, S448, S449, S450, S451, S452, S453, S454, S455, S456, S457, S458, S459, S460, S461, S462, S463, S465, S466, S467, S468, S471, S476, S477, S478, S482, S483, S484, S485, S488, S490, S491, S512, S513, S519, S522, S524, S525, S546, S549, S550, S551, S552, S553, S556, S557, S558, S559, S560, S561, S562, S563, S564, S700, S701, S702, S709, S711, S714, S715, S716, S717, S718, S719, S720, S721, S722, S723, S724, S725, S726, S727, S728, S729, S730, S731, S732, S733, S734, S735, S736, S737, S738, S739, S740, S741, S742, S743, S744, S745, S747, S748, S749, S750, S751, S760, S761, S762, S764, S765, S766, S767, S770, S771, S775,

		S776, S778, S780, S781, S782, S783, S784, S805, S806, S807, S808, S810, S811, S812, S813, S815, Z552, Z553, Z569, Z737, Z750
Excluded concurrent procedures	CCI	1RB52BA, 1RB52CQ, 1RB57DA, 1RB59DAAG, 1RB59DAAN, 1RB59DAGX, 1RB59LAGX, 1RB87DA, 1RB87LA, 1RB87RA, 1RB89DA, 1RB89LA, 1RD89DA, 1RD89LA, 1RD89RA, 1RF50BAGX, 1RF51FJAL, 1RF51FJFA, 1RF51FJLV, 1RF59BAGX, 1RF87LA, 1RF87RA, 1RF89LA, 1RM53BAEM, 1RM53CABH, 1RM53CAEM, 1RM53DAEM, 1RM57CAGX, 1RM59DAAG, 1RM87DAAG, 1RM87DAGX, 1RM87LAGX, 1RM89AA, 1RM89CA, 1RM89DA, 1RM89LA, 1RM91LA, 1RN53CAEM, 1RN55CAEB
Tubal ligation	CCI	1RF51DAAL, 1RF51DAFA, 1RF51DAFF, 1RF51DALV, 1RF51FJFF, 1RF51FJGE, 1RF51LAAL, 1RF51LAFA, 1RF51LAFF, 1RF51LALV, 1RF55DAFF, 1RF59BAAG, 1RF59DAAG, 1RF59DAGX, 1RF59LAAG, 1RF59LAGX, 1RF87DA, 1RF87LA, 1RF89DA, 1RF89LA, 1RF89RA
	CCP	7800, 7810, 7820, 7821, 7822, 7830, 7831, 7832, 7839, 7840, 7841, 7842, 7849, 7850, 7851, 7852, 7853, 7859
Genitourinary complication	CCI	1PG50BA, 1PG50DA, 1PG50LA, 1PG80, 1PG82, 1PM80
Fistula	CCI	1NP86MH, 1PM86GH, 1PM86MD, 1PM86MH, 1PM86RA, 1PM86RB, 1PQ86MH, 1RS86CAXXE, 1RS86LAXXE, 1RS86MB
Gastrointestinal complication	CCI	1NK76DN, 1NK76DP, 1NK76DQ, 1NK76DR, 1NK76DS, 1NK76RE, 1NK76RF, 1NK76RJ, 1NK76SK, 1NK76SL, 1NK77EM, 1NK77EN, 1NK77RQ, 1NK77RR, 1NK80DA, 1NK80LA, 1NK82DN, 1NK82DP, 1NK82EM, 1NK82EN, 1NK82RE, 1NK82RF, 1NK82RQ, 1NK82RR, 1NK87BA, 1NK87DA, 1NK87DN, 1NK87DP, 1NK87DX, 1NK87DY, 1NK87LA, 1NK87RE, 1NK87RF, 1NK87TF, 1NK87TG, 1NM76DF, 1NM76DN, 1NM76RE, 1NM76RN, 1NM77EP, 1NM77RS, 1NM80DA, 1NM80LA, 1NM82DF, 1NM82EP, 1NM82RN, 1NM82RS, 1NM87BA, 1NM87DA, 1NM87DE, 1NM87DF, 1NM87DN, 1NM87DX, 1NM87DY, 1NM87LA, 1NM87PN, 1NM87RD, 1NM87RE, 1NM87RN, 1NM87TF, 1NM87TG, 1NP72DA, 1NP72LA, 1NQ72DA, 1NQ72LA, 1NQ72PB, 1NQ80LA, 1NQ80PB, 1NQ80PF
Pain	ICD-10	R102, R1039, R104
Control of bleeding	CCI	1RM13CA, 1RM13GQ, 1RN13CA, 1RS13CA, 1RS13GQ
Blood transfusion	CCI	1LZ19HH, 1LZ19HM
Infection complication	ICD-10	A4188, A419, J129, J13, J14, J15, J16, J17, J18, L022, L023, L028, L029, L032, L0330, L0331, L0332, L0333, L0335, L0336, L0339, L038, L039, N300, N302, N308, N309, N70, N71, N72, N730, N731, N732, N733, N734, N735, N738, N739, N743, N744, N748, R572, R650, R651, R652, R653, R659, T814
Venous thromboembolism	ICD-10	I260, I269, I822, I823, I828, I829
Fluid overload	ICD-10	E871, E877, E878, G936
Thermal injury	ICD-10	M6135, N765, N766, T240, T241, T242, T243, T282, T283, T284

Other injury	ICD-10	K65, K660, K661, N837, N856, N857, N858, N859, N882, N990, N991, N992, N994, N998, N999, R100, S3084, S3085, S3140, S3150, S355, S357, S358, S359, S36450, S36451, S36460, S36461, S36490, S36491, S36500, S36501, S36510, S36511, S36590, S36591, S36600, S36601, S36610, S36611, S36690, S36691, S37100, S37101, S37110, S37111, S37190, S37191, S37200, S37201, S37210, S37211, S37290, S37291, S37300, S37301, S37310, S37311, S37390, S37391, S37400, S37401, S37410, S37411, S37490, S37491, S37500, S37501, S37510, S37511, S37590, S37591, S37600, S37601, S37610, S37611, S37690, S37691, S37700, S37701, S37710, S37711, S37790, S37791, S37800, S37801, S37810, S37811, S37890, S37891, S37900, S37901, S37910, S37911, S37990, S37991
